# Supplementary material for: Tetrapod sperm length evolution in relation to body mass is shaped by multiple trade-offs
Source: Nat Commun. 2024 Jul 22;15:6160. doi: 10.1038/s41467-024-50391-0 (PMC11263692; doi:10.1038/s41467-024-50391-0)
Supplement: Supplementary file 1 — Supplementary Information [file 41467_2024_50391_MOESM1_ESM.pdf]

Supplementary Information

Tetrapod sperm length evolution in relation to body mass is shaped by multiple trade-offs

Koçillari L.[1,2,3§], Cattelan S.[4,5§*], Rasotto M.B.[4,6], Seno F.[2], Maritan A.[2,6], Pilastro A.[4,6]

[1] Istituto Italiano di Tecnologia, 38068 Rovereto, Italy

[2] Department of Physics and Astronomy, Section INFN, University of Padova, 35131 Padova, Italy

[3] Institute for Neural Information Processing, Center for Molecular Neurobiology Hamburg (ZMNH), University Medical Center Hamburg-Eppendorf (UKE), D-20251 Hamburg, Germany

[4] Department of Biology, University of Padova, 35121 Padova, Italy

[5] Fritz Lipmann Institute – Leibniz Institute on Aging, 07745 Jena, Germany

[6] National Biodiversity Future Center, 90133 Palermo, Italy

§ these authors contributed equally to this work

Correspondence to: silvia.cattelan@leibniz-fli.de

This Supplementary Information file contains:

- Supplementary Figures page 2
- Supplementary Methods pages 3-7

Supplementary Figures

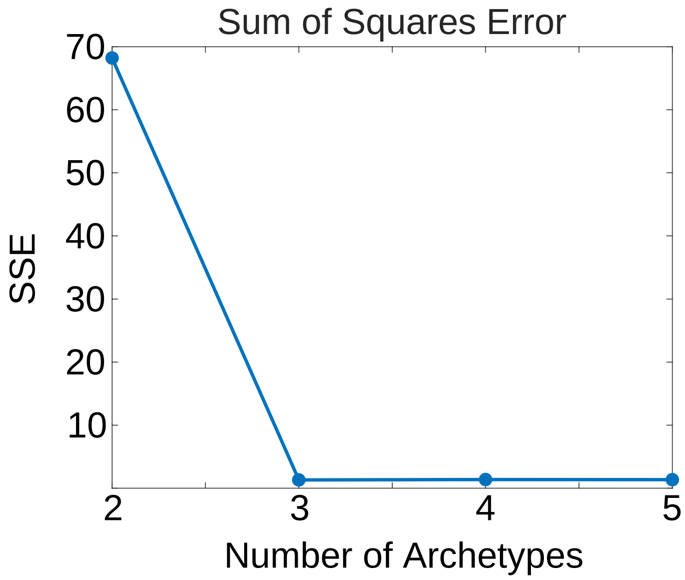

Supplementary Figure 1. Sum of squares error as a function of the number of vertices. Sum of squares error was computed with the PCHA algorithm. Three is the optimal number of vertices that minimizes the errors. Then, a plateau is reached.

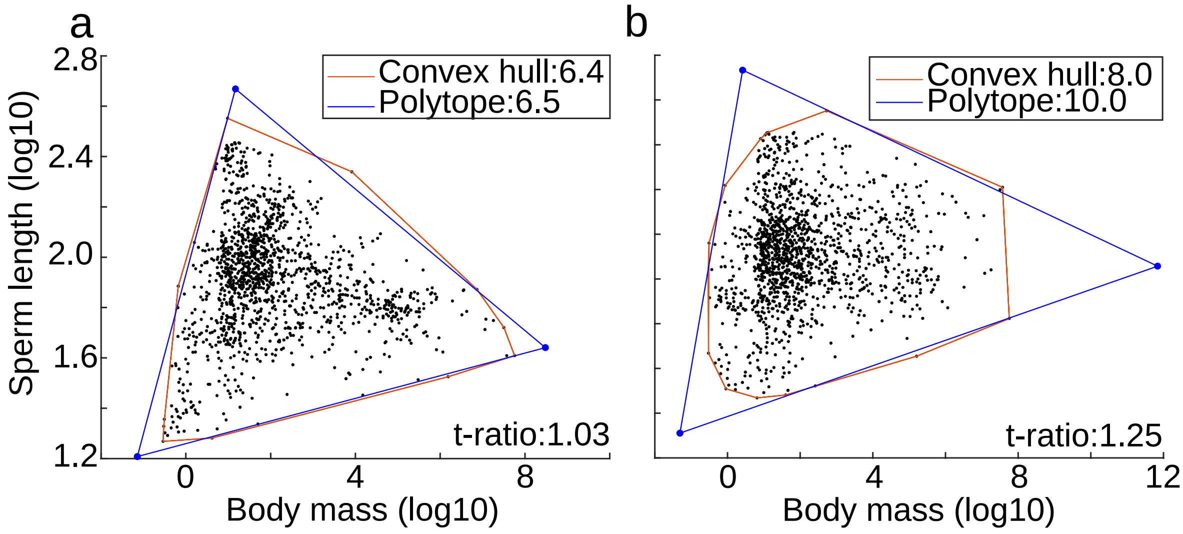

Supplementary Figure 2. T-ratio test to determine the significance of the triangular Pareto front. a. Data points are shown as black dots. The red polygon corresponds to the best convex hull that encapsulates all the data points and was computed using the "convhulln" algorithm in MATLAB . The blue polygon represents the best-fitted triangle that encloses most data points but leaves some outliers outside. We computed it via the Sisal algorithm[1]. **b.** Randomized data points with the SibSwap approach to correct for the phylogenetic influences. The t-ratio defines the fraction between the area of the best-fitted polytope and the area of the convex hull.

Supplementary Methods

Data collection

All data and the associated references are reported in the dataset uploaded in Figshare (https://figshare.com/articles/dataset/Dataset_Tetrapod_sperm_length_evolution_in_relation_to_body_mass_is_shaped_by_multiple_trade-offs_/26022289). During data collection, we prioritized data from recent sources that contained data for multiple species, and supplementing data for individual species when possible. To avoid conflicts among datasets, we followed a standardized protocol when collecting data. Firstly, we preferred sources that reported measures from n>1 individual and we thus excluded values measured from a single individual or from dead animals. We considered only mean values instead of maximal values. Then we preferred to not include values collected using data extraction software from images and values collected without a described and standardized methodology (e.g., "personal observations"). If multiple sources fit the above criteria, we prioritized more recent values. Where multiple data remained, we prioritized the dataset with the largest sample size. In a few cases we spotted clear errors in the values reported in the most recent study and we thus decided to report the value contained in the original reference. All the species names were uniformed to the most recent nomenclature or in the case of equivalent synonymous, the most used were chosen. For Amphibia, we limited our analysis to anurans species, thus excluding two orders: Urodela and Gymnophiona. While we did not find data relative to Gymnophiona species, we decided to exclude Urodela (salamanders) for two reasons: 1) on average their sperm are extremely long and significantly longer than the average sperm size of tetrapods[2] and 2) we did not found data on clutch size and testes mass for the salamanders in our dataset. Using salamander species for exploring sperm size-body mass morphospace would have extended the distribution of sperm size without the possibility to perform enrichment analysis on those phenotypes given the absence of clutch size and testes mass data. We also excluded an outlier for sperm size: an anuran species (*Discoglossus pictus*) in which males produce the longest vertebrate sperm measured (2.5 mm)[2]. It is worth noting, however, that the exclusion of *Discoglossus pictus* and Urodela species did not significantly affect the shape of the morphospace given by the sperm size and body mass.

Relative testes size as an index of the level of sperm competition

Testes mass has been shown to closely reflect the level of sperm competition among species and, within species, between males with alternative mating strategies[3]. Since testes mass is also strongly correlated with body mass both in our dataset (Supplementary Fig. 3) and in other published studies (e.g. see Fig. 1 in[4]), residuals of the log-log linear regression of testes mass on body mass are routinely used to estimate whether the testes of a species are larger (reflecting high levels of sperm competition) or smaller (reflecting lower levels of sperm competition) than expected for the body size in comparative studies. Citing[3]: *"This prediction [i.e. that testes size reflects sperm competition level] is so widespread that testes size (correcting for body size) is commonly used as a proxy of sperm competition, even in the absence of any other information about a species' reproductive behaviour"*. We therefore used the same approach to provide results that are comparable with previous research in the field. Since tetrapod classes have significantly different regression slopes[4] (Supplementary Fig. 3), we calculated body mass-corrected testes size (hereafter RTS) from class-specific regression lines (Supplementary Table 1).

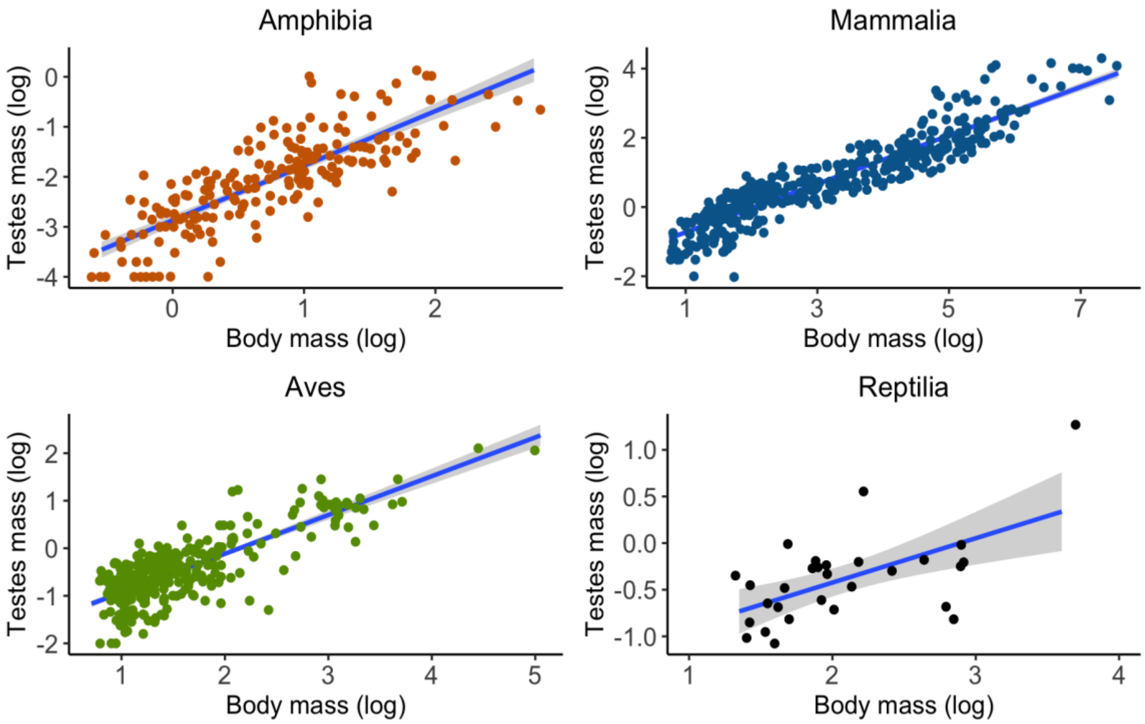

Supplementary Figure 3. Relationship of body mass with testes mass. Each class of tetrapods are plotted in the log-log space of body mass and testes mass. Amphibia n=197, Mammalia n=408, Aves n=324, Reptilia n=29.

Supplementary Table 1. Results from log-log linear regression of testes mass on body mass in our sample of tetrapod classes.

Class	intercept	SE	b	SE	*beta*	t	p	n
**Amphibia**	-2.868	0.056	1.092	0.056	0.813	51.04	8.87E-115	197
**Aves**	-1.747	0.056	0.816	0.032	0.815	31.39	5.32E-100	324
**Mammalia**	-1.430	0.051	0.700	0.015	0.921	27.80	5.18E-96	408
**Reptilia**	-1.376	0.272	0.476	0.126	0.587	5.06	2.58E-05	29

We tested the standardized residuals (RTS) of class-specific regressions for normality and outliers (i.e. residuals that were >3 standard deviations from the mean distribution). Visual inspection of Q-Q plots (Supplementary Fig. 4) demonstrated a nearly perfect fit between observed and expected RTS and identified very few possible outliers (Amphibia n=1, Aves n=3, Mammalia n=4, and none for Reptilia). We then re-calculated the log-log regressions after excluding the identified outliers and compared the residuals without outliers ($RTS_{outliers}$) with the original RTS values. In all three classes, the correlation between RTS and $RTS_{outliers}$ was > 0.999 (Supplementary Table 2).

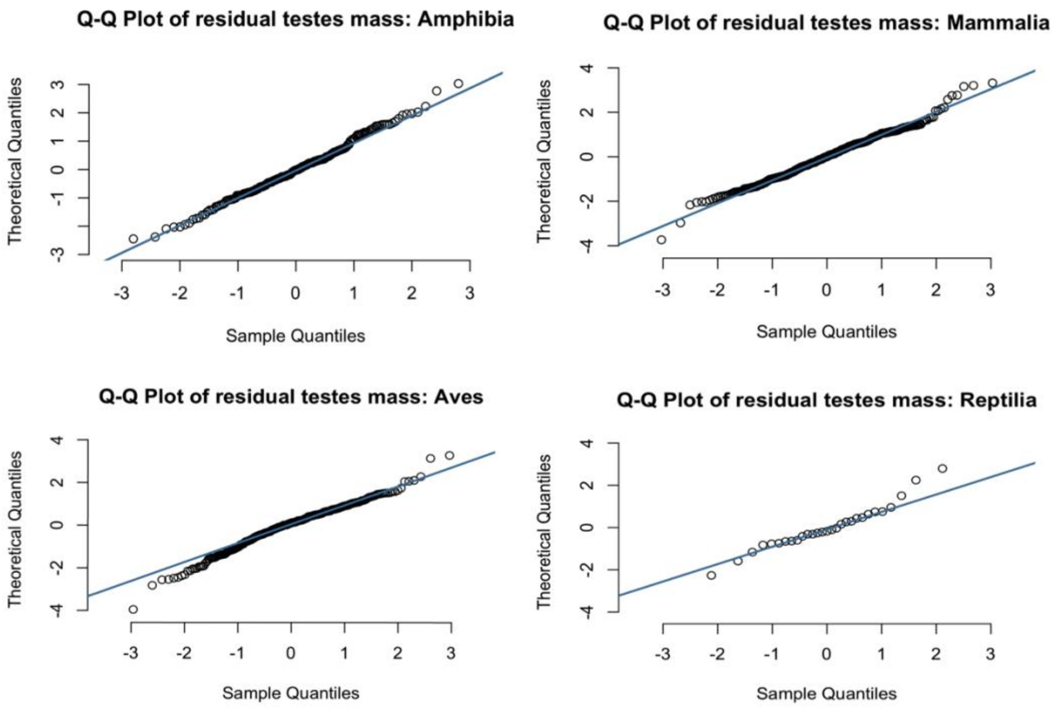

Supplementary Figure 4. Q-Q plots of observed distribution of RTS values vs expected distribution. Values >3 standard deviations are considered as outliers.

Supplementary Table 2. Correlation between RTS calculated on the entire dataset and after the exclusion of the outliers.

Class	r	n	outliers
**Amphibia**	0.999957	196	1
**Aves**	0.999996	321	3
**Mammalia**	0.998966	404	4

Robustness of the Pareto front: control for phylogenetic bias between 65 Mya and the present day

Our control for phylogenetic biases indicates that the triangular-shaped Pareto front remained robust to phylogenetic dependencies until approximately 65 Mya, at which point the p-value exceeded the 0.05 threshold (Fig. 5d). To investigate the loss of significance at around 65 Mya, we hypothesized that the SibSwap randomization approach might become overly conservative as we approach the terminal nodes because each sibling tip contains less and less species as we get closer to the present day. In such case, the SibSwap-shuffled distributions could lead to nonsignificant outcomes when compared to the original Pareto front (p-values > 0.05), because the actual distributions of species might closely resemble those of the shuffled distributions. We therefore reasoned that in such scenarios, a potential solution might be to consider the species within each sibling tip as an independent data point in the trait space by calculating the average value. The distribution of the obtained average values can be then tested within a triangular region in the trait space, as predicted by the Pareto theory. Importantly, the randomized datasets would now shuffle traits across all average values, which are treated as independent data points in the trait space. First, the number of sibling tips ranged from 68 to 922 as we approached the terminal nodes of the tree (Supplementary Fig. 5a). We then checked the marginal distributions of the traits in the sibling tips (Supplementary Fig. 5b) and we noticed that the marginal distributions of the species within the sibling tips resembled those of the original dataset. Subsequently, we calculated the centroids of each sibling tip by taking the averages for each trait across species within the sibling tip and plotted the centroids in the trait space. When evaluating the triangularity of the distributions as a function of time points, we found that the p-values were mostly significant, indicating that the average values generated a continuously filled triangular distribution in trait space (Supplementary Fig. 5c). In

summary, our results suggest that regardless of whether we considered old phylogenetic relationships among species (355 Mya – 65 Mya), or recent phylogenetic relationships (below 65 Mya), there is no strong phylogenetic bias affecting the significance of the triangular-shaped Pareto front.

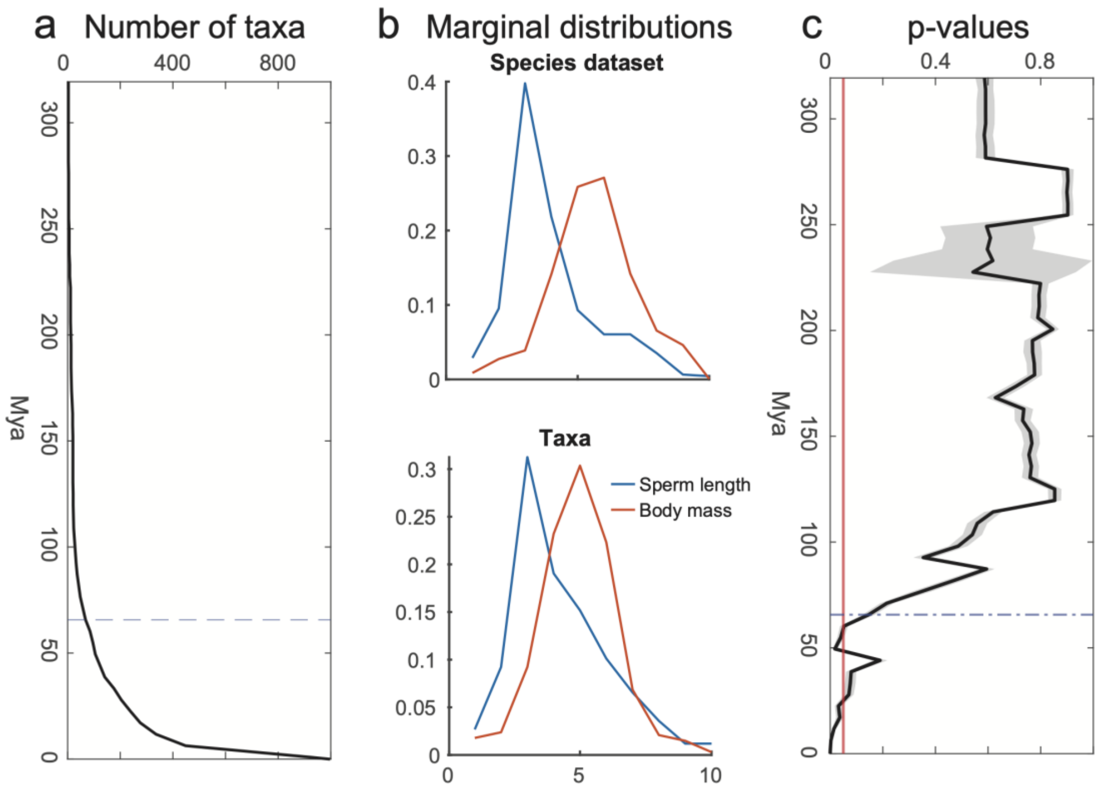

Supplementary Figure 5. Robustness of the Pareto front: control for phylogenetic bias in the averaged groups. a. Number of clusters. **b.** Marginal distributions of sperm length and body mass in the actual dataset and in an example of clusters obtained after averaging species into clusters at around 330 Mya. **c.** We computed the p-values based on the t-ratio test using the randomization across all clusters at different time points. Mean and shaded areas represent the mean and standard deviation across 100 iterations respectively. The vertical red line corresponds to p-value = 0.05, while the blue dashed line marks the line between not significant phylogenetic dependencies (until 65Mya) and significant phylogenetic dependencies (from 65 Mya to the present). Statistical comparisons were performed using a one-sample one-sided t-test.

Supplementary References

1 Bioucas-Dias, J. M. A variable splitting augmented Lagrangian approach to linear spectral unmixing. *2009 First Workshop on Hyperspectral Image and Signal Processing: Evolution in Remote Sensing*, 1-4, doi:10.1109/WHISPERS.2009.5289072 (2009).

2 Pitnick, S., Hosken, D. J. & Birkhead, T. R. *Sperm morphological diversity (in Sperm Biology: An Evolutionary Perspective)*. (Academic Press, 2009).

3 Lüpold, S., de Boer, R. A., Evans, J. P., Tomkins, J. L. & Fitzpatrick, J. L. How sperm competition shapes the evolution of testes and sperm: a meta-analysis. *Philosophical transactions of the Royal Society of London. Series B, Biological sciences* **375**, 20200064, doi:10.1098/rstb.2020.0064 (2020).

4 Baker, J., Humphries, S., Ferguson-Gow, H., Meade, A. & Venditti, C. Rapid decreases in relative testes mass among monogamous birds but not in other vertebrates. *Ecol. Lett.* **23**, 283-292, doi:https://doi.org/10.1111/ele.13431 (2020).
