# Supplementary material for: Tetrapod sperm length evolution in relation to body mass is shaped by multiple trade-offs
Source: Nat Commun. 2024 Jul 22;15:6160. doi: 10.1038/s41467-024-50391-0 (PMC11263692; doi:10.1038/s41467-024-50391-0)
Supplement: Supplementary file 3 — Reporting summary [file 41467_2024_50391_MOESM3_ESM.pdf]

nature portfolio

Corresponding author(s): Silvia Cattelan

Last updated by author(s): June, 12 2024

Reporting Summary

Nature Portfolio wishes to improve the reproducibility of the work that we publish. This form provides structure for consistency and transparency in reporting. For further information on Nature Portfolio policies, see our Editorial Policies and the Editorial Policy Checklist.

Statistics

For all statistical analyses, confirm that the following items are present in the figure legend, table legend, main text, or Methods section.

n/a	Confirmed	
☐	☒	The exact sample size (*n*) for each experimental group/condition, given as a discrete number and unit of measurement
☐	☒	A statement on whether measurements were taken from distinct samples or whether the same sample was measured repeatedly
☐	☒	The statistical test(s) used AND whether they are one- or two-sided<br>*Only common tests should be described solely by name; describe more complex techniques in the Methods section.*
☐	☒	A description of all covariates tested
☒	☐	A description of any assumptions or corrections, such as tests of normality and adjustment for multiple comparisons
☐	☒	A full description of the statistical parameters including central tendency (e.g. means) or other basic estimates (e.g. regression coefficient) AND variation (e.g. standard deviation) or associated estimates of uncertainty (e.g. confidence intervals)
☐	☒	For null hypothesis testing, the test statistic (e.g. *F*, *t*, *r*) with confidence intervals, effect sizes, degrees of freedom and *P* value noted<br>*Give P values as exact values whenever suitable.*
☒	☐	For Bayesian analysis, information on the choice of priors and Markov chain Monte Carlo settings
☒	☐	For hierarchical and complex designs, identification of the appropriate level for tests and full reporting of outcomes
☒	☐	Estimates of effect sizes (e.g. Cohen's *d*, Pearson's *r*), indicating how they were calculated

Our web collection on statistics for biologists contains articles on many of the points above.

Software and code

Policy information about availability of computer code

Data collection	We did not use any code for collecting the data.
Data analysis	The code used for data analysis can be found here on Github: https://github.com/lorenkocillari/Pareto_Sperm_Length_Evolution and it is also deposited in Zenodo (doi: 10.5281/zenodo.11621774). For running the code we used Matlab version 2021a

For manuscripts utilizing custom algorithms or software that are central to the research but not yet described in published literature, software must be made available to editors and reviewers. We strongly encourage code deposition in a community repository (e.g. GitHub). See the Nature Portfolio guidelines for submitting code & software for further information.

Data

Policy information about availability of data

All manuscripts must include a data availability statement. This statement should provide the following information, where applicable:

- Accession codes, unique identifiers, or web links for publicly available datasets
- A description of any restrictions on data availability
- For clinical datasets or third party data, please ensure that the statement adheres to our policy

All data and the associated references are reported in the dataset uploaded in Figshare (https://figshare.com/articles/dataset/Dataset_Tetrapod_sperm_length_evolution_in_relation_to_body_mass_is_shaped_by_multiple_trade-offs_/26022289)

Research involving human participants, their data, or biological material

Policy information about studies with human participants or human data. See also policy information about sex, gender (identity/presentation), and sexual orientation and race, ethnicity and racism.

Reporting on sex and gender	NA
Reporting on race, ethnicity, or other socially relevant groupings	NA
Population characteristics	NA
Recruitment	NA
Ethics oversight	NA

Note that full information on the approval of the study protocol must also be provided in the manuscript.

Field-specific reporting

Please select the one below that is the best fit for your research. If you are not sure, read the appropriate sections before making your selection.

☐ Life sciences ☐ Behavioural & social sciences ☒ Ecological, evolutionary & environmental sciences

For a reference copy of the document with all sections, see nature.com/documents/nr-reporting-summary-flat.pdf

Ecological, evolutionary & environmental sciences study design

All studies must disclose on these points even when the disclosure is negative.

Study description	We aimed at testing whether the sperm length-body mass relationship in tetrapods results from trade-offs between multiple tasks. We collected 1388 tetrapod species for which at least data on sperm length and body mass were available. We then explored the three main factors predicted to influence sperm size evolution, namely sperm competition, clutch size and genome size. We tested both the entire tetrapod dataset as a whole and within subgroups based on thermoregulation mode (endothermic versus ectothermic tetrapods), fertilization mode (internal versus external fertilizer species) and at the class level.
Research sample	We analyzed a dataset of 1388 tetrapods (Mammalia n=643, Aves n=399, Amphibia n=231, Reptilia n=115) and for each species collected a maximum of 5 trait values, namely sperm size (µm), body mass (g), clutch/litter size, relative testes mass (as a proxy for sperm competition), and genome size. We collected sperm size data from the most comprehensive published dataset to date on sperm size and from other sources. Body mass, clutch size, testes mass, and genome size were collected from published and publicly available sources. We included only a single source per species; when more than one value for a single species was present, we prioritized the most recent one. More details on our data collection procedure can be found in the Supplementary Materials and final sample sizes for each group and trait are reported in Table 1 in the main text. We listed all the sources that we used in the dataset that we uploaded in Figshare (https://figshare.com/articles/dataset/Dataset_Tetrapod_sperm_length_evolution_in_relation_to_body_mass_is_shaped_by_multiple_trade-offs_/26022289).
Sampling strategy	Data were collected from literature as described above, and our final size resulted from the inclusion/exclusion criteria that we described above.
Data collection	During data collection, we prioritized data from recent sources that contained data for multiple species, and supplementing data for individual species when possible. To avoid conflicts among datasets, we followed a standardized protocol when collecting data. Firstly, we preferred sources that reported measures from n>1 individual and we thus excluded values measured from a single individual or from dead animals. We considered only mean values instead of maximal values. Then we preferred to not include values collected using data extraction software from images and values collected without a described and standardized methodology (e.g."personal observations"). If multiple sources fit the above criteria, we prioritized more recent values. Where multiple data remained, we prioritized the dataset with the largest sample size. In a few cases we spotted clear errors in the values reported in the most recent study and we thus decided to report the value contained in the original reference. All the species names were uniformed to the most recent nomenclature or in the case of equivalent synonymous, the most used were chosen. Andrea Pilastro and Silvia Cattelan initially collected all the data. Silvia Cattelan generated the final dataset with all the associated sources by following the standardized protocol described above.
Timing and spatial scale	Data were collected between July 2017 and August 2021.
Data exclusions	For Amphibia, we limited our analysis to anurans species, thus excluding two orders: Urodela and Gymnophiona. While we did not find data relative to Gymnophiona species, we decided to exclude Urodela (salamanders) for two reasons: 1) on average their sperm are extremely long and significantly longer than the average sperm size of tetrapods1 and 2) we did not found data on clutch size and testes mass for the salamanders in our dataset. Using salamander species for exploring sperm size-body mass morphospace would have extended the distribution of sperm size without the possibility to perform the enrichment analysis on those phenotypes given the absence of clutch size and testes mass data. We also excluded an outlier for sperm size: an anuran species (Discoglossus pictus)

	in which males produce the longest vertebrate sperm measured (2.5 mm1). It is worth noting that the exclusion of Discoglossus pictus and Urodela species did not significantly affect the shape of the morphospace given by the sperm size and body mass.
Reproducibility	The results can be reproduced by running the code we provided. Importantly, we also tested for the reproducibility of our analysis by randomly shuffling the values of either sperm length or body mass across phylogenetically close species while keeping unchanged the values of the other trait. This approach allowed us to test for phylogenetic bias.
Randomization	No randomization was necessary in this study.
Blinding	NA

Did the study involve field work? ☐ Yes ☒ No

Reporting for specific materials, systems and methods

We require information from authors about some types of materials, experimental systems and methods used in many studies. Here, indicate whether each material, system or method listed is relevant to your study. If you are not sure if a list item applies to your research, read the appropriate section before selecting a response.

Materials & experimental systems

n/a	Involved in the study
☒	☐ Antibodies
☒	☐ Eukaryotic cell lines
☒	☐ Palaeontology and archaeology
☒	☐ Animals and other organisms
☒	☐ Clinical data
☒	☐ Dual use research of concern
☒	☐ Plants

Methods

n/a	Involved in the study
☒	☐ ChIP-seq
☒	☐ Flow cytometry
☒	☐ MRI-based neuroimaging

Plants

Seed stocks	NA
Novel plant genotypes	NA
Authentication	NA
